# The Role of the Bisphenol A in Diabetes and Obesity

**DOI:** 10.3390/biomedicines9060666

**Published:** 2021-06-10

**Authors:** Marcelino Pérez-Bermejo, Irene Mas-Pérez, Maria Teresa Murillo-Llorente

**Affiliations:** 1 SONEV Research Group, School of Medicine and Health Sciences, Catholic University of Valencia San Vicente Mártir, C/Quevedo nº 2, 46001 Valencia, Spain; mt.murillo@ucv.es; 2 School of Medicine and Health Sciences, Catholic University of Valencia San Vicente Mártir, C/Quevedo nº 2, 46001 Valencia, Spain; imaspe@mail.ucv.es

**Keywords:** Bisphenol A, obesity, endocrine disruptor, glucose intolerance, insulin resistance

## Abstract

Bisphenol A is a compound commonly found in products meant for daily use. It was one of the first compounds to be identified as an endocrine disruptor that was capable of disrupting the endocrine system and producing very similar effects to those of metabolic syndrome. It has recently gained popularity in the scientific arena as a risk factor for obesity and diabetes due to its ability to imitate natural oestrogens and bind to their receptors. The aim was to study the possible relationship between the Bisphenol A endocrine disruptor with diabetes and obesity. The analysis of the articles allows us to conclude that Bisphenol A is an additional risk factor to consider in the development of diabetes and obesity, since it is capable of stimulating the hypertrophy of adipocytes and altering the endocrine system by mimicking the effects of the oestrogen molecule, since epidemiological studies carried out have suggested that the same disruptions seen in experimental studies on animals can be found in humans; however, despite many countries having developed policies to limit exposure to this disruptor in their populations, there is a lack of international agreement. Understanding its relationship with obesity and diabetes will help to raise awareness in the population and adopt public health campaigns to prevent exposure—especially among young people—to these substances.

## 1. Introduction

Obesity is a chronic metabolic disease affecting people of all ages and is one of the 21st century’s major public health problems, which the WHO considers to be an epidemic that affects both adults and the child-youth population [1]. Increased calorie consumption and decreased physical activity are the main risk factors in developing obesity. However, this imbalance between energy intake and expenditure should not be considered the only reasons for the large increase in obesity rates we find today. As with other chronic health problems, obesity and being overweight are the result of a complex interaction between genetic, behavioural, and environmental factors. While evidence is strong for some of these contributors, for others, it is still emerging. The massive marketing and consumption of foods that are high in calories and a lack of physical activity are accepted as the main culprits for this epidemic, although we must remain sceptical and not assume that they are the only ones or that they are sufficient to explain it adequately.

Many studies that link calorie consumption with obesity are based on subjective questionnaires completed by the study’s participants themselves and have been shown to be inaccurate on numerous occasions, mainly due to reporting errors and the variation in calorie intake from one day to another [2]. In 2015, experts from the Energy Balance Measurement Working Group published a study [3] whereby they concluded that future studies should have these subjective questionnaires replaced or linked to other more objective methods such as the measurement of biomarkers, as many of them have been linked to obesity. For example, some hormones, such as leptin or resistin, cytokines, such as visfatin, or plasma proteins, such as haptoglobin, have been shown to be good predictors for the level of adiposity. On the other hand, good predictors for obesity and insulin resistance are C-reactive protein, sialic acid, plasminogen activation inhibitor 1, and the Von Willebrand factor [4]. Biological markers are currently increasingly used because they have been shown to be more accurate than questionnaires [5].

Since the theory that the imbalance between calorie contribution and intake is not enough in explaining the current high rates of obesity, there is increasing evidence to support the hypothesis that environmental chemicals around us—particularly those that are capable of interacting with the endocrine system—known as endocrine disruptors (ED), are an additional risk factor to take into account [6,7]. Bisphenol A or BPA was one of the first compounds to be identified as an obesogenic endocrine disruptor along with pesticides, heavy metals, and polychlorinated bisphenols (PCBs). BPA is considered an ED due to its ability to act as an oestrogen in some tissues [8,9].

Several international organisations have defined endocrine disruptors, but the most widely used definition seems to be that by the European Union, which indicates that an ED is an “exogenous substance that causes adverse health effects in an intact organism, or its progeny, secondary to changes in endocrine function” [10]. Due to its ability to secrete adipokines, adipose tissue is considered an endocrine organ and may be the target of EDs.

Most EDs are man-made compounds—they can be found in food, everyday products, or the environment—and are capable of accumulating in the human body [9,11]. Understanding the relationship between EDs and obesity would help to raise awareness in the population and the adoption of public health campaigns to prevent exposure to these substances, especially among the youngest in society, given that several recent epidemiological studies suggest that rapid weight gain during childhood could be related to maternal or early extrauterine life of the newborn exposure to ED [12,13,14,15,16].

Numerous epidemiological studies have found a positive link between BPA levels in urine and obesity and diabetes in adults [15,16] and in children [17,18]. Most epidemiological studies into the relationship between BPA and obesity or diabetes do so through levels of this compound found in urine, as BPA is not stored in the body; it has a half-life of six hours and is excreted in urine [19]. The issue with epidemiological studies in this context is that most studied the relationship of each ED individually without giving consideration to people being exposed to several different EDs and that they may interact with each other or with other factors. This is why this literature review focuses on articles that study the mechanisms of this relationship, trying to analyse how BPA disrupts glucose metabolism and insulin resistance to clarify the true role of BPA in the development of obesity and diabetes.

### 1.1. Epidemiology of Obesity and Diabetes

The prevalence of obesity has been increasing steadily since the mid-20th century, accelerating rapidly since the 1980s [20]. In the most developed countries, this increase has occurred across a large part of the population, including a great variety of ages, races, sex, and socio-economic levels [20,21]. The number of overweight or obese youths has tripled in the last three decades, increasing faster than obesity rates in adults in most countries [22]. It is currently estimated that 8% of children between 4 and 5 years old in the United States are overweight according to the National Health and Nutrition Examination Survey, a research programme carried out by the National Center for Health Statistics [20].

In Spain, obesity rates are one of the highest according to the Organisation for Economic Co-operation and Development (OECD). One in three children between the ages of 13 and 14 is overweight, and one in six adults is obese [20,23]. According to the most recent data from the 2017 National Health Survey, the obesity rate in men has multiplied by 2.3 and in women by 2.4 in the last 30 years; increasing from 7.9% to 18.2% and from 6.9% to 16.7% of the population, respectively. Regarding the figures of those who are overweight, the difference between men and women is much greater: 44.31% in men compared to 30.04% in women in 2017 [24]. As well as the high prevalence rates, it has been witnessed that disorders associated with obesity, such as metabolic syndrome, a precursor to type 2 diabetes, are already present early in life [25]. There is also evidence that childhood obesity is a precursor to obesity in adulthood [26].

Diabetes is a chronic disease that occurs when the pancreas does not produce enough insulin or when the body does not use the insulin effectively. The effect of uncontrolled diabetes is hyperglycaemia (increased blood sugar), which over time seriously damages many organs and systems, especially nerves and blood vessels [27,28]. Type 2 diabetes mellitus (DM2) is the most frequent form of presentation, accounting for 90% of the total cases. Type 1 diabetes mellitus (DM1) cases represent 5 to 10% of total cases and the remaining percentage corresponds to other forms of presentation. The prevalence is increasing rapidly due to the improvement in life expectancy and changes in lifestyle habits [28,29]. In 2014, this disease was suffered by 8.5% of adults and 1.6 million individuals died from diabetes. Since then, its prevalence has increased most significantly in low- and middle-income countries. In 2018, the global prevalence of DM2 among adults was approximately 415 million, but according to projections from the International Diabetes Federation, it is expected to reach 642 million by 2040 [30,31].

### 1.2. Bisphenol A

Bisphenol A or BPA is what is known as a xenoestrogen, an endocrine disruptor that interferes with the endocrine system by imitating the effects of natural oestrogens and that is able to keep the endocrine system in a constant state of flux given that they are widely incorporated in many areas of modern society today. Due to its ability to disrupt the endocrine system, developmental periods, including the prenatal period and infancy, are critical periods in terms of sensitivity to the effects of BPA [32]. It belongs to the chemical group of phenols and is a synthetic compound made up of polycarbonate polymer units and epoxy resins. It is widely used in a wide variety of products—plastic, aluminium, or polycarbonate containers—and has an oestrogenic action [33]. Animal and human research has associated BPA with many health problems, including infertility, weight gain, behavioural changes, early-onset puberty, prostate and mammary gland cancers, cardiovascular effects, obesity, and diabetes [34,35].

#### 1.2.1. Oestrogenic Characteristics of BPA

**Figure 1:**
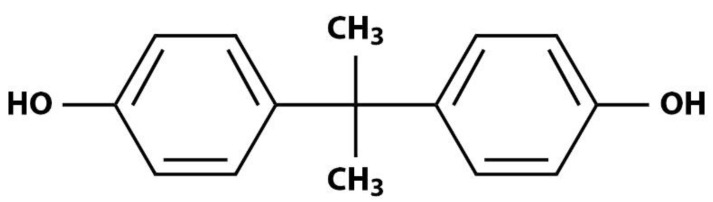
Chemical structure of bisphenol A.

BPA has a characteristic structure that gives it the ability to mimic oestrogens by binding to their receptors. It is formed by two groups of hydroxy-phenols connected to a carbon atom with the formula (CH3)2C(C6H4OH)2 (Figure 1) and it is specifically in the hydroxy-external groups that it is similar to the oestrogenic molecule 17β-oestradiol (E2). BPA has a mass of 228.3 Da, while oestrogen has a mean mass of 272.4 Da [36].

Until relatively recently, BPA was considered a weak oestrogen. In some studies from 2003, the response of animal tissue to the presence of BPA was 10,000 to 100,000 times less potent than that produced by oestrogen [8,37]. However, more recently it has been possible to see the mechanisms at the molecular level by which BPA can elicit a response in cells at very low concentrations due to its binding to the nuclear oestrogen receptors alpha (ER-α) and beta (ER-β) [38]. Although this binding is relatively weak, around two times less potent than a physiological oestrogen molecule, the fact that exposure to BPA is chronic and continuous must be taken into account [39]. BPA, however, as with other xenoestrogens, is capable of displacing radioactively labelled oestrogen from both its ER-α and ER-β [39]. Other authors have studied different mechanisms by which BPA could exert its endocrine disruptor activity. These mechanisms occur by means of signalling pathways that are bound to membrane and non-nuclear receptors, such as those previously discussed [38,40].

Alonso-Magdalena et al. [41] showed how pancreatic cells react in a similar way when exposed to similar concentrations of environmental BPA or to physiological concentrations of oestrogen. BPA regulates the concentration of pancreatic insulin through a mechanism that involves the activation of ER-α and they, therefore, concluded that environmental BPA produces the same response as endogenous oestrogen in pancreatic cells. Another important conclusion they made is that ER-α is the main receptor involved in regulating insulin content for BPA and oestrogen. Other work carried out several years later also found a similar response in vivo when treating mice with small doses of BPA for four days and—after this time—finding increased insulin in pancreatic cells with this response also being dependent on ER-α [42].

Due to the discovery of the effects BPA has on health, there are alternatives today, such as bisphenol AF (BPAF), bisphenol B (BPB), bisphenol F (BPF), bisphenol S (BPS), and 4-cumylphenol (HPP). These compounds are gradually replacing BPA in some plastics. In any case, there are still only a few studies regarding the effect of these compounds on the endocrine system, but these analogues have already been found in various food products, such as juices, dairy products, oils, and fish, among others, using the high-performance liquid chromatography-tandem mass spectrometry (HPLC-MS/MS) [43]. Conversely, some studies have confirmed that they do have oestrogenic activity in vitro, and that, consequently, it is possible that they can disrupt the endocrine system in a manner similar to that of BPA [44,45,46].

#### 1.2.2. Sources and Levels of Exposure to BPA in Humans

The main sources of exposure to BPA include water bottles, food packaging, infant feeding bottles, toys, thermal paper, household appliances, dental materials, and medical equipment [11,47,48,49,50]. A recent paper [51] has published an extensive list of products that contain BPA, grouping them into food/beverages, electronic equipment, paper products, textile samples, electrical equipment, medical devices, and other.

Despite its rapid elimination, the pervasive presence of BPA in our environment means that humans are continuously exposed to it. Metabolic disorders or dysfunctions caused in adipocytes have been described in animal studies and epidemiological studies as well as in vitro studies and, although the capacity this substance may have to disrupt the endocrine system is increasing, disagreement between researchers continues. Furthermore, the effects of BPA on obesity are not only measured using BMI but also in the effects it produces on lipids, glucose, and adipose tissue.

Several studies have shown that BPA migrates from food and drink containers to the contents [47,52,53]. The Canadian Food Research Division found concentrations of BPA in all canned non-alcoholic drinks, but not in those in glass containers, indicating that BPA migration from the container to the content is higher in canned drinks [47]. Goodson et al. [52] established that BPA migration from aluminium containers to drinks occurs during the processing time and does not increase once this process is finished, which seems to indicate that BPA residues are capable of passing to the content of the container at high temperatures such as those that occur during the processing time but not at room temperature once this stage has completed.

In addition to contamination by mouth, another well-known source of BPA is heat-sensitive paper, which is used for purchase receipts in supermarkets, shops or cash machines. Several articles have shown that the BPA present in this material could be transferred to the skin when held for no more than 5 s and that it remains detectable after 2 h, indicating its passage to under the skin [54,55].

Humans are constantly exposed to BPA at such a level that it can be considered an environmental factor. With more than 2.7 million tonnes of BPA produced in 2003 and 4.5 million in 2015, BPA is one of the most abundant environmental chemicals [38]. BPA production is increasing the fastest in East Asia—predominantly China—with a trend from 59% in 2010 to 68% in 2015. However, the trend has been decreasing in Europe from 32% in 2010 to 25% in 2015. A similar trend is seen in the United States with consumption down from 28% in 2010 to 18% in 2015 [56].

BPA concentrations of 11.2 ng mL^−1^ have been detected in urine in the placenta [57], 4.4 ng mL^−1^ in the umbilical cord [58], 8.3 ng mL^−1^ in amniotic fluid [59], 3.4 ng mL^−1^ in the colostrum, and 7.3 ng mL^−1^ in breast milk [60]. These findings are proof that BPA concentrations transfer from mother to child, either through the placenta or breast milk. Another source of newborns’ exposure to BPA is through baby bottles. A study carried out by Brede et al. [61] showed that all the newly purchased bottles released small concentrations of BPA of between 0.11 to 0.43 µg^−1^ into water and that after about 50 washes, they found BPA levels that were higher than the first time with concentrations varying between 3.7 and 17 µg^−1^. Taking into account that BPA exposure in the general population is virtually unavoidable due to its wide-ranging presence in the plastics of food containers or water bottles, measures should be taken to limit their exposure, especially to children and pregnant women. Many countries in Europe are trying to reduce exposure by banning the use of BPA in food packaging. As an example, there is a new law in France that will ban the use of plastics in contact with food in all school canteens [62].

After several studies showed the harmful effects of BPA during the earliest stages of life, France banned the use of BPA from the plastics used in baby bottles. This ban was later extended to all of Europe and many countries took steps to limit the use of BPA in food packaging, especially after the European Food Safety Authority (EFSA) defined the maximum limit for BPA intake at 4 µg/kg of body weight per day [63]. However, not all countries have taken similar measures. In the USA, for example, although the use of BPA in baby bottles was banned, an evaluation project published in 2014 reported that the dose at which no related side effects derived from BPA were observed was 5 mg/kg of body weight per day [64]. Therefore, despite many health authorities in the world enacting policies to limit exposure to BPA in their populations, especially in children, there is a certain lack of international agreement regarding the non-harmful limit of exposure to BPA. This disagreement, as the WHO mentions, is due in part to the lack of adequate experimental studies in animals for population risk assessment, and also to controversial differences in the results of different studies [65].

#### 1.2.3. The Role of BPA in the Disruption of Glucose Metabolism and Insulin Resistance

The ability of BPA to interfere with the endocrine system by binding to oestrogen receptors allows it to cause an imbalance similar to the endocrine disruption that occurs during pregnancy due to changes in oestrogen levels, such as the disruption in glucose metabolism and insulin resistance (IR) [66]. One of the functions of oestrogens is involvement in regulating the energy balance, glucose homoeostasis, and insulin sensitivity. Both aforementioned oestrogen receptors—ER-α and ER-β—are present in the hypothalamic nucleus, but ER-α has been shown to be the main isoform in terms of controlling the body’s energy balance [67].

**Figure 2:**
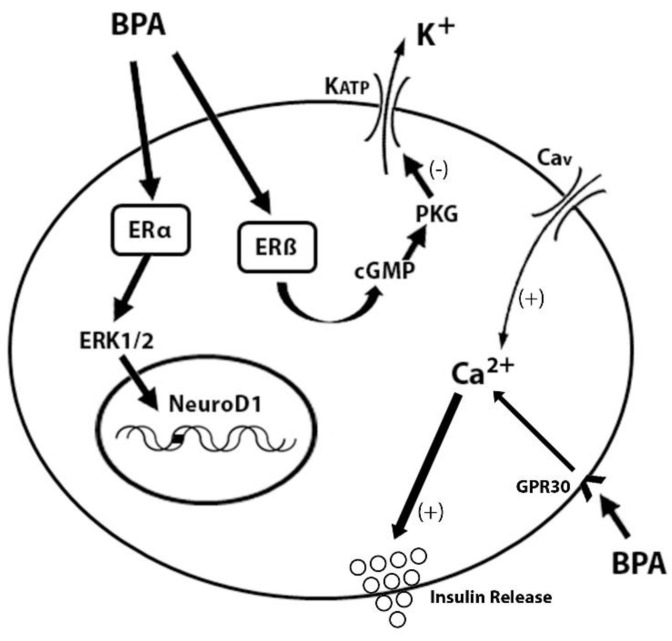
Mechanism of the action of BPA through intracellular ERα and ERβ receptors and those present in the plasma membrane of the β cell (GPR30). Modified from [75].

Several studies [68,69,70,71] have shown that BPA binds to membrane-bound forms of the ER (mER) and with high affinity to a transmembrane ER receptor GPR30. In addition to its oestrogenic activity, there is mounting evidence that BPA interacts with other nuclear receptors, although at higher concentrations, for example, it binds to the thyroid hormone receptor (TR) with lower affinity than the ER [72]. In pancreatic β-cells, in the presence of stimulatory glucose concentrations, low concentrations of BPA produce the activation of the PKG by the cGMP, and rapidly decrease the KATP channel activity through ERβ, enhancing glucose-induced [Ca^2+^] i signals and insulin release. In addition, there is an activation of the transcription of the insulin gene via ERα—ERK1/2 that activates the transcription factor NeuroD1 (See Figure 2) [41,73,74].

Gestational insulin resistance is a natural phenomenon that appears physiologically during pregnancy to more easily direct nutrients present in blood to the growing foetus [61,76]; however, plasma glucose levels can increase and promote gestational diabetes. Insulin resistance specifically appears as the result of a hormonal action originated by the placenta. Jointly with insulin resistance, glucose metabolism is also disturbed—manifested through the attenuation of glucose uptake by muscle, adipose, and liver tissue and inadequate suppression of gluconeogenesis in the liver [77]. The fact that glucose uptake by maternal tissues decreases implies an increase in blood glucose levels, which is beneficial for the embryo’s nutrition. A 2016 study suggested that placental lactogen promotes the consumption of lipids in the mother’s body more so than glucose, and this also contributes to preserving glucose for the embryo’s nutrition [78]. Later, as an adaptation mechanism to these physiological disruptions, the body increases insulin production by using pancreatic β cells. Another adaptation mechanism occurs in pancreatic β cells located in the islets of Langerhans; these undergo hypertrophy and start to secrete greater amounts of insulin. In some cases where the pancreatic β cells do not respond well to changes in the body, the ineffectiveness of this adaptation mechanism leads to gestational diabetes, shown by an increase in blood glucose levels in previously healthy women with no history of diabetes. The pancreatic β cells express both oestrogen and progesterone receptors, and in gestational diabetes they may stop responding adequately to stimuli from these hormones [75]. The oestrogen molecule has the ability to bind to a membrane receptor and close the K^+^ channels in it. The membrane then depolarises, which allows calcium to enter that triggers the release of insulin. In a study carried out by a group of researchers at Miguel Hernández University in Alicante, it was shown that the exposure of adult mice to a low dose (10 μg/kg) of E2 (17-β-oestradiol) or BPA induced an increase in plasma insulin. They also saw that longer exposures induced an increase in the insulin content in pancreatic ß cells in response to a stimulus of their oestrogen receptors. They began to see these effects at doses as low as 10 μg/kg and from day two. After several days of treatment with E2 or BPA, the mice developed chronic hyperinsulinaemia [74].

A similar effect can be observed after taking hormonal contraceptives with oestrogens, which is why they have been associated with changes in carbohydrate metabolism and increased insulin resistance [79]. Some studies show a 43–61% increase in plasma glucose in women taking this type of contraceptive [79,80], while other studies—in addition to this study—also found an increase in insulin levels during fasting and after glucose intake, and recommended that oestrogen levels in contraceptives be reduced to minimise their diabetogenic effect [79,80].

Several studies have analysed the risk of exposure to low doses of BPA on the metabolism in animals, finding hyperinsulinaemia, lower body temperatures, and lower physical activity in exposed mice compared to the control group [81]. When exposure was combined with a high-fat diet during pregnancy, high levels of fasting blood glucose, glucose intolerance, and high levels of non-esterified fatty acids in plasma were found in the offspring compared to the control group [82].

Exposure during pregnancy in women causes glucose intolerance and high levels of insulin, triglycerides, and leptin in plasma compared to the control group, which seems to indicate that exposure to BPA during pregnancy promotes glucose intolerance [83]. The long-term consequences that were observed were weight gain for at least four months, increased perigonadal fat, decreased insulin sensitivity, and elevated plasma insulin levels compared to the control group; the elevation of leptin levels remained for several months [84,85].

Other studies showed that exposure to BPA can cause increased adipogenesis in human cells. Their results indicated that it is not just BPA, but also its main metabolite that is capable of stimulating adipocyte differentiation [86,87]. Exposure to BPA appears to decrease adipocyte sensitivity to insulin due to decreased expression of glucose transporter 1 (GLUT1) and phosphorylation of the insulin receptor, suggesting that BPA also induces disorders in the metabolism of glucose and increases the risk of type 2 diabetes [86,87,88,89].

The lack of consensus on the safety of BPA combined with the fact that more and more articles have indicated that exposure to BPA is directly related to an increased risk of causing endocrine system disruptions drove us to carry out this literature review to better understand the mechanisms by which endocrine disruptors, specifically BPA, disrupt the molecular pathways of the endocrine system.

## 2. Search Methodology

The literature search was carried out on PubMed and Web Of Science. The search strategy was carried out by combining the MeSH terms ‘bisphenol A’, ‘BPA’, ‘endocrine disruptors’, ‘obesity’, ‘insulin resistance’, and ‘glucose intolerance’, combined with each other using Boolean operators. Articles from within the last 10 years were selected, giving a total of 17 to review. All the articles included the Bisphenol A endocrine disruptor and its relationship with insulin resistance or glucose intolerance. They have been divided into two groups for review and analysis. On the one hand, those dealing with experimental research studies in animals (Table 1) and, on the other, observational studies in humans (Table 2). In all experimental works, the intervention consisted of exposure to doses of BPA. In observational studies, the relationship between the levels of BPA in urine or blood with disruptions in glucose and/or insulin metabolism was studied.

## 3. Discussion and Conclusions

There is more and more evidence to point to endocrine disruptors being an additional risk factor to take into account. Understanding the relationship between them and obesity or diabetes would help ensure that appropriate measures are taken to raise awareness among the population and to prevent their negative effects on health.

In recent decades, endocrine disruptors have been subject to much research and debate, and the amount of information about them today is overwhelming. There are many articles dealing with endocrine disruptors and the trend is for publications on them to increase given that it is a fairly recent topic with many unresolved issues. Conversely, as indicated above, phenols are widely-used compounds in modern society in developed countries and are increasingly being used in developing countries. This means that science cannot ignore whether one of the many properties of this compound is to disrupt the endocrine system. The numerous studies published in recent years—both experimental and epidemiological—have contributed to our understanding of some of the properties and to better understand how they affect people.

Some studies have shown how adipocytes can hypertrophy when exposed to certain concentrations of BPA as occurs in obesity, as well as presenting a higher prevalence of obesity, abdominal obesity, and insulin resistance [15,95,96,102]. The effects of BPA on obesity are not only measured using BMI, but also in the effects it produces on lipids, glucose, and adipose tissue.

The result of this review suggests that bisphenol A is capable of acting as an endocrine disruptor through the modifications produced by this chemical in glucose and insulin homoeostasis [81,92], specifically when this exposure occurs at low BPA concentrations [90,97,98] and during the foetal period [91,93,94]. The explanation most often proposed by the scientific community to explain this phenomenon lies in the xenoestrogenic properties of this compound. Due to a certain structural similarity between BPA and 17-beta-oestrogen molecules, the former can bind to the oestrogen receptors of pancreatic beta cells that have an insulin-stimulating action.

The tissues most susceptible to BPA are related to embryonic development (such as the placenta, umbilical cord, and amniotic fluid) as well as postnatal development under maternal influence (such as breast milk and colostrum). It is, therefore, logical that experimental studies in mice showed transgenerational effects in offspring, even though they have not been exposed to BPA. Increased insulin secretion stands out among these effects, which is the body’s physiological response to carbohydrate intolerance, which occurs in metabolic syndrome and type 2 diabetes.

In addition to observing that offspring had increased insulin secretion compared to the control groups, other arguments supporting the role of BPA as an endocrine disruptor are that the levels of leptin, triglycerides, and glycerol also increased [83]. These findings are similar to those found in metabolic syndrome, one of the main causes being obesity. Metabolic syndrome is considered a disease in itself that also increases the risk of type 2 diabetes and cardiovascular diseases. One of the main conclusions drawn from this review is that BPA is capable of disrupting the endocrine system, producing effects that are very similar to those of metabolic syndrome, such as elevated triglyceride levels and insulin resistance.

One of the current unanswered questions about BPA is at what concentrations its effects occur and whether there is a limit beyond which its ability as an endocrine disruptor decreases. Alonso-Magdalena et al. and Wei et al. obtained similar conclusions when it was observed that the disruptions mainly occurred at low doses of BPA and not at higher doses. This suggests that there may be a critical narrow concentration range for the action of BPA and that exposures above or below that range would be less harmful to the body [83,95].

Several authors have analysed what happens in prolonged foetal exposure to BPA during the neonatal period and have evaluated the metabolic disruptions that exposure to environmentally equivalent concentrations normally received by humans in mice and their offspring could have and concluded that females exposed to BPA during childhood showed signs of obesity and metabolic disturbances such as increased triglyceride levels, hyperinsulinaemia, and insulin resistance [83,91,92,93,95,99]. Other studies, however, showed glucose metabolism disruptions to be greater in males [95,98]. This is another discrepancy that continues to exist among researchers. Studies pursuing this specific objective would be required to clarify whether there is a difference between the effect produced in men and women, as currently, there does not appear to be sufficient evidence to support one position or the other. The reason why some studies have found differences between sexes still remains unclear; the main difference found is that males have greater insulin resistance than females. One of the proposed explanations lies in a different hepatic metabolism of BPA. Females have been found to have higher levels of UDP-glucuronosyltransferase, which is involved in the transformation of BPA and its elimination from the body [103].

The disruptions produced by BPA in glucose metabolism and insulin homoeostasis can also occur physiologically in humans at the time of gestation, in which a physiological or pathological insulin resistance occurs, as in type 2 diabetes. Nutrition plays a key role in this second case, which is why the effects of prenatal BPA exposure on adipose tissue and metabolism in goats were analysed, introducing a novel factor of analysing whether a high-calorie, high-fat diet would exacerbate the disruptions caused by BPA. The combination of both factors was not seen to increase endocrine disruptions; nevertheless, they did find that the disruptions produced by both were similar: insulin resistance and adipocyte hypertrophy [96]. The results of all these studies in animals show how metabolic disruptions in pancreatic cells and adipocytes are similar to those in obesity.

In the second part of the results of this literature review, we analysed articles that studied the levels of BPA in humans [15,100,101,102,103]. Most of these epidemiological studies do so by measuring the concentration in urine, as the half-life of BPA is very short and its excretion is in urine, which makes measuring its concentration in urine easier than in blood. All studies in this literature review found a positive relationship, but some have weak evidence as they excluded other factors that may influence the metabolic disruptions caused by BPA. Several of these studies highlighted the triple association between BPA, obesity, and diabetes due to the ability of BPA to mimic natural oestrogens in the body [15,102]. Due to a certain structural similarity between the 17- β-oestradiol and BPA molecules, this can bind to the oestrogen receptors of pancreatic beta cells that have an insulin-stimulating action.

The results obtained from these studies add to the conclusions already drawn from experimental studies in animals. In pre-adolescent females exposed to BPA, the baseline levels of oestradiol and androstenedione were significantly higher than in those who were not exposed, with the elevated levels of these hormones and greater insulin resistance remaining one year later [100]. This suggests the exposure to BPA in pre-adolescents can disrupt endocrine metabolism due to its ability to act as a natural oestrogen.

Although concentrations of BPA are more difficult to measure in blood due to its short half-life, many participants have also presented with disrupted basal blood glucose levels and insulin resistance [102]. We also found discrepancies in the differences between sexes. Wang et al. concluded that exposure to BPA was associated with impaired glucose homoeostasis prior to development of diabetes in middle-aged and elderly women, but not in men [101].

The main limitation of this work is that most of the articles analysed are animal studies and not human studies due to the logical ethical difficulties of carrying out this type of study in humans. Another limitation is that the disruptions in the endocrine system produced by bisphenol A have been studied without considering possible interactions with other external factors.

As indicated above, there is not a general consensus of all countries in the establishment of a limit as a safe dose, so as not disrupt the glucose-stimulated insulin response in humans [51,63,64,103]. It is appropriate to keep this information in mind for prevention, as this compound is widely used in many countries and avoiding its exposure is tricky. Today, BPA-free plastic products are increasingly available, such as BPA-free water bottles, but the use of BPA-free polymers is not widespread and BPA is often simply replaced by one of its analogues, BPS or BPF, which may exhibit similar behaviours as BPA. Some of the current alternatives are to replace it with other materials that do not have an oestrogenic activity, such as glass or plastics for bottles or food containers without phenols.

In conclusion, endocrine disruptors may be an additional risk factor to consider in the development of obesity as they are capable of stimulating adipocyte hypertrophy and this appears to confirm a positive association between the levels of BPA in the body and obesity. The results of the experimental studies mostly point to BPA having the ability to disrupt the endocrine system by mimicking the effects of the oestrogen molecule, which is why more experimental and epidemiological research will be necessary to establish the scale of the effects caused by BPA in large populations and its association with insulin resistance and diabetes. Epidemiological studies carried out on humans suggest that the same disruptions seen in experimental studies on animals may be found; however, despite many countries having developed policies to limit exposure to BPA in their populations, there is a lack of international agreement. Understanding the relationship between EDs and obesity will help to raise awareness in the population and adopt public health campaigns to prevent exposure—especially among young people—to these substances.

## Figures and Tables

**Table 1:** Experimental studies in animals.

Ref.	Year	No. of Subjects	Exposure Dose	Stage	Conclusions
[81]	2012	2 groups 6–9 mice/group	100 µg/kg body weight/day	3-month-old male OF-1 mice	The mice exposed to BPA developed insulin resistance and hyperinsulinaemia and increased secretion in response to glucose stimulation. They also presented a lower body temperature and were less active than those in the control group.
[83]	2010	3 groups 8–13 mice/group	10 or 100 μg/kg body weight/day	3-month pregnant OF-1 mice	BPA produces metabolic disorders that disrupt glucose homoeostasis, which is considered a risk factor for diabetes. The offspring has lower glucose tolerance, as well as higher insulin resistance and higher plasma levels of insulin, leptin, triglycerides, and glycerol.
[90]	2013	7 groups 13–17 mice/group	5, 50, 500, 5000, and 50,000 μg/kg body weight/day	3-month-old nulliparous female CD-1	The mice exposed to BPA at four different doses significantly lose the ability to maintain normal glucose levels and show greater insulin insensitivity.
[91]	2015	3 groups 9–13 mice/group	lower dose: 10 μg/kg body weight/day; upper dose: 10 mg/kg body weight/day	Six-week-old virgin C57BL/6 females	Early life exposure to environmental EDs can disrupt the metabolism of the developing foetus, as well as that of its offspring. Weight gain was observed in male offspring compared to controls, as well as glucose intolerance during adulthood.
[92]	2019	3 groups 11 mice/group	lower dose: 10 μg/kg body weight/day; upper dose: 10 mg/kg body weight/day	C57BL/6J virgin female mice (F0) crossed with 8–10 weeks old C57BL/6J males	This study suggests that BPA exposure can be passed down through generations by epigenetic modifications. The third generation has a lower number of pancreatic ß cells and an increase in insulin secretion.
[93]	2016	Control group: 73 mice; BPA10 group: 63; BPA100 group: 56.	10 or 100 μg/kg body weight/day	3-month pregnant OF-1 mice	The offspring of pregnant females exposed to BPA at doses of either 10 μg/kg/day or higher doses of 100 μg/kg/day presented higher levels of insulin in the blood compared to the control group.
[94]	2016	Not Available	5 mg/kg body weight/day	6-8-week-old breeding pairs of adult C57BL/6 mice	Exposure to BPA increases glucagon expression and the number of ß-cell islets in the pancreas that express it, suggesting that BPA may disrupt pancreatic cell differentiation.
[95]	2011	64 mice (32 female and 32 male)	50, 250, or 1250 μg/kg body weight/day. Dosages were adjusted daily for body weight changes of pregnant rats (2.0 mL/kg body weight)	Virgin female (270–300 g) and male (350–400 g) genitor Wistar rats. Pups measured on postnatal days 1, 5 10, 15, and 21	Perinatal exposure to BPA is implicated in the development of obesity and compromises the proper metabolism functioning, particularly when exposure is at small doses (50 ug/kg). The males of the offspring present hyperinsulinaemia and metabolic disturbances that increase with age.
[96]	2016	Groups of 6–9 female sheep	0.05, 0.5, or 5 mg/kg body weight/day from days 30 through 90 of gestation	Adult Suffolk breed sheep (2–5 y old)	Exposure to BPA during foetal life at levels equivalent to those found in humans can disrupt metabolism, leading to insulin resistance and adipocyte hypertrophy. The defects produced in the metabolism of glucose and insulin are similar to those produced by a high-fat diet.
[97]	2012	Not Available	1 nM	Adult C57 female mice	Environmentally significant doses of BPA have an insulinotropic action on the islets of Langerhans.
[98]	2013	15–30 mice per group	100 µg/kg body weight/day	8 weeks old male and female C57BL6 mice (F0)	The results suggest that exposure to BPA could contribute to the appearance of metabolic disorders that lead to significant disruptions in glucose homoeostasis. Furthermore, the effects of BPA seem to be dose-, sex-, and time-dependent and are greater if exposure occurs during the foetal development period.
[99]	2017	15–30 mice per group	0, 0.25, 2.5, 25, or 250 μg BPA/kg body weight/day from GD 8 to lactational day 16	Breeding pairs of adult C57BL/6 mice	The effects of BPA are dose- and sex-dependent. The second exposure exacerbated the adverse effects of BPA exposure in females, who presented signs of obesity and metabolic disturbances such as increased triglyceride levels, hyperinsulinaemia, and insulin resistance.

OF-1, CD-1, C57BL/6 they are species of mice.

**Table 2:** Observational studies in humans.

Ref.	Year	Subjects	Conclusions
[15]	2012	3390 aged 40 y or older	Levels of BPA in urine are positively associated with obesity, increased abdominal fat, and insulin resistance in Chinese adults and the elderly.
[100]	2013	48 children aged 7 to 8 years	Exposure to BPA in pre-adolescents can disrupt endocrine metabolism due to its ability to act as a natural oestrogen. In the group exposed to BPA, the levels of base oestradiol and androstenedione were significantly higher than in the control group. A year later, the girls who had been exposed to BPA showed elevated levels of these hormones as well as insulin resistance.
[101]	2019	2336 aged 40 y or older followed for 4 years	Exposure to BPA was independently associated with impaired glucose homoeostasis prior to development of diabetes in middle-aged and elderly women, but not in men. In women, increased urinary concentrations of BPA were associated with an increased risk of developing hyperglycaemia and dysfunction of pancreatic ß cells.
[102]	2015	76 Caucasian male 53.5 ± 5.7 mean y age	In subjects with higher levels of BPA in the blood, higher levels of inflammation markers were found, and they had higher percentages of visceral fat and higher metabolic syndrome and insulin resistance prevalence.
[103]	2018	8 healthy adult males and 8 adult females (postmenopausal), with obesity and prediabetes by HbA1c	This study suggests that exposure to BPA at a dose of 50 ug/kg may disrupt the glucose-stimulated insulin response in humans. A strong positive relationship is found between HbA1c and the percentage changes in the insulin index.
